# Genetic evidence supports a distinct lineage of American crocodile (*Crocodylus acutus*) in the Greater Antilles

**DOI:** 10.7717/peerj.5836

**Published:** 2018-11-12

**Authors:** Yoamel Milián-García, Michael A. Russello, Jessica Castellanos-Labarcena, Martin Cichon, Vikas Kumar, Georgina Espinosa, Natalia Rossi, Frank Mazzotti, Evon Hekkala, George Amato, Axel Janke

**Affiliations:** 1 Departamento de Bioquímica, Facultad de Biología, Universidad de La Habana, La Habana, Cuba; 2 Department of Biology, University of British Columbia, Kelowna, BC, Canada; 3 Senckenberg Biodiversity and Climate Research Centre, Senckenberg Gesellschaft für Naturforschung, Frankfurt am Main, Germany; 4 Key Laboratory of Vertebrate Evolution and Human Origins, Institute of Vertebrate Paleontology and Paleoanthropology, Chinese Academy of Sciences, Beijing, China; 5 Wildlife Conservation Society, New York, NY, USA; 6 Fort Lauderdale Research and Education Center, University of Florida, Fort Lauderdale, FL, USA; 7 Department of Biological Sciences, Fordham University, New York, NY, USA; 8 Sackler Institute for Comparative Genomics, American Museum of Natural History, New York, NY, USA

**Keywords:** *Crocodylus acutus*, American crocodile, mtDNA, Phylogeny, Cryptic species

## Abstract

Four species of true crocodile (genus *Crocodylus*) have been described from the Americas. Three of these crocodile species exhibit non-overlapping distributions—*Crocodylus intermedius* in South America, *C. moreletii* along the Caribbean coast of Mesoamerica, and *C. rhombifer* confined to Cuba. The fourth, *C. acutus*, is narrowly sympatric with each of the other three species. In this study, we sampled 113 crocodiles across *Crocodylus* populations in Cuba, as well as exemplar populations in Belize and Florida (USA), and sequenced three regions of the mitochondrial genome (D-loop, cytochrome b, cytochrome oxidase I; 3,626 base pair long dataset) that overlapped with published data previously collected from Colombia, Jamaica, and the Cayman Islands. Phylogenetic analyses of these data revealed two, paraphyletic lineages of *C. acutus*. One lineage, found in the continental Americas, is the sister taxon to *C. intermedius*, while the Greater Antillean lineage is most closely related to *C. rhombifer*. In addition to the paraphyly of the two *C. acutus* lineages, we recovered a 5.4% estimate of Tamura-Nei genetic divergence between the Antillean and continental clades. The reconstructed paraphyly, distinct phylogenetic affinities and high genetic divergence between Antillean and continental *C. acutus* populations are consistent with interspecific differentiation within the genus and suggest that the current taxon recognized as *C. acutus* is more likely a complex of cryptic species warranting a reassessment of current taxonomy. Moreover, the inclusion, for the first time, of samples from the western population of the American crocodile in Cuba revealed evidence for continental mtDNA haplotypes in the Antilles, suggesting this area may constitute a transition zone between distinct lineages of *C. acutus*. Further study using nuclear character data is warranted to more fully characterize this cryptic diversity, resolve taxonomic uncertainty, and inform conservation planning in this system.

## Introduction

The order Crocodylia is currently comprised of 27 recognized or proposed living species distributed among three families (Alligatoridae, Crocodylidae, and Gavialidae) and nine genera (*Alligator*, *Caiman*, *Melanosuchus*, *Paleosuchus*, *Crocodylus*, *Mecistops*, *Osteolaemus*, *Tomistoma*, and *Gavialis*) representing, along with birds, the unique surviving members of the Archosauria (Grigg & Kirshner, 2015). The genus *Crocodylus* alone comprises almost 50% (12 out of 27) of all extant crocodylian species and is unique for its global distribution in the tropics and recent diversification (Oaks, 2011). Yet, ambiguity remains regarding the phylogenetic relationships within the genus *Crocodylus* (Meganathan et al., 2010; Man et al., 2011; Meredith et al., 2011; Hekkala et al., 2011; Oaks, 2011; Shirley et al., 2014). For example, the sister relationships of the four New World *Crocodylus* species have not been consistent and significantly vary depending on the origin of the species sampled (Milián-García et al., 2015, 2018).

Cuba represents an important area in the distribution of New World *Crocodylus*, given its geographic proximity to mainland North America (∼150 km from Florida, USA) and Central America (∼210 km from the Yucatán peninsula, Mexico) and the fact that it hosts two *Crocodylus* species. The Cuban crocodile (*Crocodylus rhombifer*), endemic to the main island of Cuba, is largely restricted to Zapata Swamp (Matanzas Province) where it is sympatric with *C. acutus*. In most other coastal areas in Cuba, including Birama Swamp in the southeast and the Guanahacabibes´ peninsula (Pinar del Río province) on the western tip, *C. acutus* is found in allopatry with no evidence of historical overlap with *C. rhombifer* (Tabet et al., 2014). Previous studies have indicated that the phylogenetic placement of *C. acutus* is sensitive to sampling location, with most studies relying solely on individuals sampled from continental populations in Central and/or South America (Meredith et al., 2011; Milián-García et al., 2011, 2015; Oaks, 2011). In these cases, a sister taxon relationship between *C. acutus* and *C. intermedius* has been consistently recovered with high support (Brochu & McEachran, 2000; Meganathan et al., 2010; Meredith et al., 2011; Hekkala et al., 2011; Oaks, 2011). However, studies that included Antillean *C. acutus* sampled in Cuba have revealed a well-supported sister relationship with *C. rhombifer*, to the exclusion of continental *C. acutus* that continued to cluster with *C. intermedius* (Milián-García et al., 2011, 2015, 2018). The observed inconsistency in phylogenetic placement of *C. acutus* is likely a consequence of excluding intraspecific genetic variation when trying to resolve higher order relationships.

Although Cuban and American crocodiles have been included in previous phylogenetic studies, insufficient sampling has precluded a definitive investigation of cryptic diversity in *C. acutus* and what impact that might have on phylogenetic reconstruction, taxonomic status, and conservation management. To fill this knowledge gap, here we collected mitochondrial DNA character data for *C. acutus* sampled throughout Cuba to characterize patterns of variation and lineage diversity across the island. We combined these data with exemplar sampling and previously collected data from Central America (Colombia, Belize), North America (Florida, USA) and elsewhere in the Greater Antilles (Jamaica and Cayman Islands) to reconstruct phylogenetic relationships across the species distribution and relative to all living *Crocodylus* species. Reconstructed relationships using newly combined data sets allowed us to test a hypothesis of monophyly of *C. acutus* as currently described and explore implications for current taxonomic and conservation status in Cuba and elsewhere.

## Materials and Methods

### Sampling

**Figure 1:**
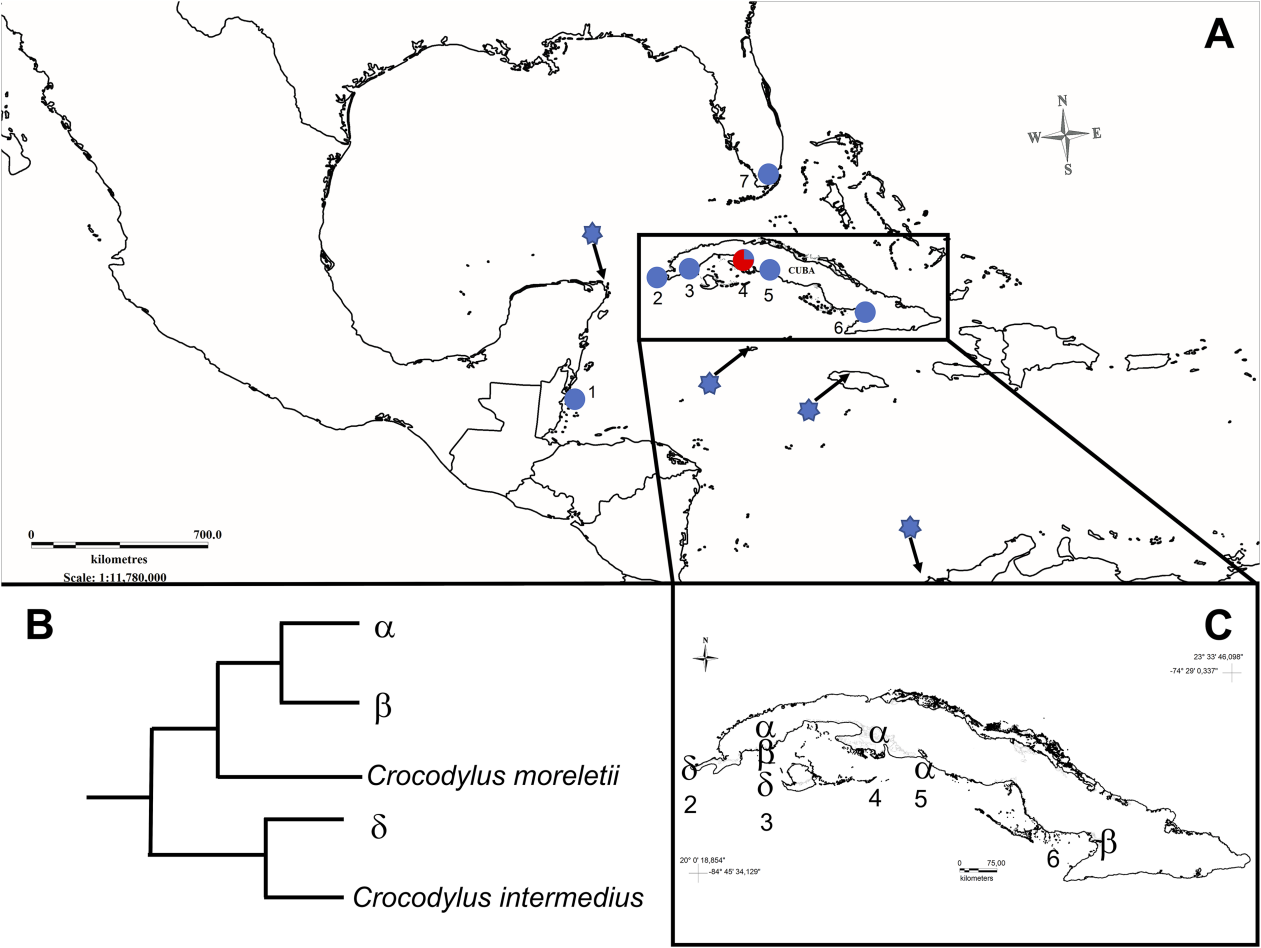
Map showing sampled populations of *Crocodylus* species in North-America, Central-America, and the Caribbean. (A) 1, Belize; 2, Guanahacabibes, Cuba; 3, Sabanalamar, Cuba; 4, Zapata Swamp, Cuba; 5, Cienfuegos, Cuba; 6, Birama Swamp, Cuba; 7, Florida, USA. Blue dots indicate *C. acutus* populations. The red and blue dot indicates the only population where *C. rhombifer* (red) and *C. acutus* (blue) were both relative proportions of samples (not exactly to scale for display purposes). The blue stars represent the reported location of the GenBank reference sequences of *C. acutus* analyzed in this study. (B) Representation of the relationships for the main mitochondrial haplotypes. (C) Distribution of main mitochondrial haplotypes across the Cuban archipelago.

We sampled a total of 65 *Crocodylus* individuals between the years 2007 and 2014 from four main areas across the Cuban archipelago (Fig. 1). These included three areas where *C. acutus* is found in allopatry to *C. rhombifer* (Birama Swamp (*n* = 27); Province of Cienfuegos (*n* = 1); Province of Pinar del Río along the Guanahacabibes peninsula (*n* = 2)) and Zapata Swamp, where the two species are sympatric and known to hybridize (*C. rhombifer* (*n* = 31); *C. acutus* (*n* = 2); suspected hybrids (*n* = 2); Milián-García et al., 2015). In addition, we sampled the most western ex situ population for *C. acutus* at the Sabanalamar captive breeding facility (*n* = 24), also located in the province of Pinar del Río. The facility was founded in 1986 with a small group of adult breeders caught in the surrounded areas of Pinar del Río, up to five individuals from Lanier Swamp, and a group of neonates from Birama Swamp (R. Rodríguez-Soberón, 2018, personal communication).

All individuals were classified as *C. rhombifer*, *C. acutus*, or as suspected hybrids based on 26 external morphological characters (Targarona, 2013) and a piece of a caudal scale was clipped from each animal’s tail for genetic analysis. We collected and transported all samples in accordance with an agreement established between the Faculty of Biology at the University of Havana and the National Enterprise for the Protection of Flora and Fauna in Cuba, and CITES export and import permits, C002135 and E-00134/14, respectively.

We also included tissue samples from continental *C. acutus* collected from the Florida Everglades, USA (*n* = 12) and Belize (*n* = 12). All of these individuals have been previously classified as *C. acutus* based on morphology and genetics (Hekkala et al., 2015; Rossi, 2016).

### Mitochondrial DNA sequencing

Total DNA was isolated for the samples from tail scale tissue preserved in 95% ethanol, using the NucleoSpin® kit for DNA extraction (Macherey-Nagel, Düren, Germany) following manufacturer’s instructions. A total of 716 base pair (bp) segment of mtDNA including the tRNAPro-tRNAPhe-D-loop region (hereafter referred to as D-loop) was amplified as a single fragment for all individuals using primers drL15459 (5′-AGGAAAGCGCTGGCCTTGTAA-3′) and CR2HA (5′-GGGGCCACTAAAAACTGGGGGGA-3′) (Weaver et al., 2008) except for individuals from the Florida Everglades and Belize. Two overlapping fragments of cytochrome b (cytb) covering a total of 1,320 bp were amplified by polymerase chain reactions (PCR) using the primers L14212 (5′-TTG GGC TTT AGACCA AGA CC-3′) with CB3H (5′-GGC AAA TAG GAA RTATCA-3′) (Palumbi, 1996), and L14849 (5′-TCCTCCACGAACGCGGAR C-3′) with H15453 (5-CCKTCCAYYTCTGTCTTACAAG-3′) (Milián-García et al., 2011). We also amplified a 665 bp fragment from the 3′ end of cytochrome oxidase I (COI) using the primer pair COIa (5′-AGT ATA AGC GTC TGGGTA GTC-3′) with COIf (5′-CCT GCA GGA GGA GGA GAY CC-3′) (Kessing et al., 1989).

We carried out the PCR on either a BioRad T100™ or an Eppendorf thermal cycler. Each PCR was prepared in a 15 μL total volume, containing: six μL of VWR® Taq DNA-polymerase (5 U/μL) Master Mix 2X, seven μL of distilled water, 0.5 μL of each primer (10 μmol), and 20–50 ng of DNA. Cycling conditions for the D-loop were as follows: 94 °C (2 min), 33 cycles of 94 °C (30 s), 58 °C (30 s), 72 °C (45 s), and a final extension of 72 °C (7 min). For cytb and COI, cycling conditions were: 94 °C (2 min), 35 cycles of 94 °C (45 s), 48 °C (45 s), 72 °C (90 s), and a final extension of 72 °C (10 min). All PCR products were purified by ExoSAP-IT (USB® Products) and sequenced using Big Dye terminators on an Applied Biosystems 3130xl Genetic Analyzer (Applied Biosystems, Foster City, CA, USA). The regions sequenced per sample are shown in Table S1.

### Data analysis

We edited and aligned mitochondrial DNA sequences in Sequencher 5.2.4 (Gene Codes, Ann Arbor, MI, USA). We also calculated haplotypic (h) and nucleotide (π) diversity (Nei, 1987) estimates based on mtDNA sequences after excluding gaps and missing data, as executed in DNASP v5 (Librado & Rozas, 2009). We then generated a haplotype network using statistical parsimony as implemented in TCS (Clement, Posada & Crandall 2000). Haplotype networks were visualized and edited using the web-based program tcsBU (Múrias dos Santos et al., 2016). In addition, we calculated Tamura-Nei genetic distances in MEGA v 7 (Kumar, Stecher & Tamura, 2016).

To examine observed haplotype variation recovered in Cuban *Crocodylus* within the context of all extant *Crocodylus*, we analyzed our sequences relative to previously published data at overlapping mtDNA fragments for all living *Crocodylus* spp. (Table 1). We tested for significant geographic structure among populations using analysis of molecular variance (AMOVA) (Excoffier, Smouse & Quattro, 1992) as implemented in ARLEQUIN v3.5 (Excoffier & Lischer, 2010). We unambiguously aligned sequences using MAFFT as implemented in GENEIOUS 10.2.4 (Biomatters Ltd., Auckland, New Zealand; Kearse et al., 2012) with default settings and concatenated the regions following their clockwise order in the mitochondrial genome (cytb-D-loop-COI). We conducted a Bayesian phylogenetic analysis for the combined dataset using MrBayes 3.0b4 (Ronquist & Huelsenbeck, 2003), assuming a mixed-model of nucleotide substitution using the best-fit model inferred for each partition separately according to the Bayesian information criterion as implemented in jModelTest 2 (Posada & Crandall, 1998). We used the African Slender-snouted crocodile (*Mecistops cataphractus*; EF551000.1) as an outgroup to root the tree. We ran four simultaneous chains for a chain length of 2 × 10^6^, each using a random tree as a starting point, the default heating scheme, and a subsampling frequency of 100. The first 2,000 trees were discarded as burn-in samples and the remaining trees were used to construct a majority-rule consensus tree and derive posterior probability values.

**Table 1:** Origin of previously published reference mtDNA sequences used per *Crocodylus* spp.

Species	GenBank accession number	Mitochondrial sequences	Authors
*C. rhombifer*	JF502247.1	Partial mitochondrial genome	Meredith et al. (2011)
*C. acutus*	HM636894.1, HQ595003, HQ595041, HQ595005, HQ595043, KF273834, KF273842, KF273841, KF273849, AY568314	Complete mitochondrial genome, Cyt b, COI, D-loop	Ray et al. (2004); Rodriguez et al., 2011; Man et al. (2011); Milián-García et al. (2011); Bloor, Ibáñez & Viloria-Lagares, (2015)
*C. moreletii*	HQ585889.1, HQ595007, HQ595045	Complete mitochondrial genome, Cyt b, COI	Meganathan et al. (2010); Milián-García et al. (2011)
*C. intermedius*	JF502242.1	Partial mitochondrial genome	Meredith et al. (2011)
*C. niloticus*	JF502245.1	Partial mitochondrial genome	Meredith et al. (2011)
*C. suchus*	JF502244.1	Partial mitochondrial genome	Meredith et al. (2011)
*C. johnsoni*	HM488008.2	Complete mitochondrial genome	Meganathan et al. (2010)
*C. porosus*	DQ273698.1	Complete mitochondrial genome	Li et al. (2007)
*C. palustris*	HM488007.1	Complete mitochondrial genome	Meganathan et al. (2010)
*C. mindorensis*	GU144287.1	Complete mitochondrial genome	Feng et al. (2010)
*C. novaeguineae*	HM636896.1	Complete mitochondrial genome	Man et al. (2011)
*C. siamensis*	EF581859.1	Complete mitochondrial genome	Srikulnath et al. (2012)

## Results

### Haplotypic variation and population differentiation

#### D-loop

The number of identified mtDNA D-loop haplotypes within Cuban *Crocodylus* ranged from one (Birama Swamp and Zapata Swamp) to four (Pinar del Río). Haplotypic and nucleotide diversities ranged from 0.000–0.634 to 0.000–0.01791, respectively, with the highest levels found in Pinar del Río, particularly the captive population (Table 2).

**Table 2:** Genetic variation within *Crocodylus* populations inferred from mitochondrial DNA markers.

Population		Mitochondrial DNA											
		D-loop				COI				Cyt b			
	*N*	*n*	No. haplotypes	Haplotype diversity	Nucleotide diversity (π)	*n*	No. haplotypes	Haplotype diversity	Nucleotide diversity (π)	*n*	No. haplotypes	Haplotype diversity	Nucleotide diversity (π)
Captive *C. acutus*-Pinar del Río	24	24	4	0.634 (0.00580)	0.01791	22	3	0.558 (0.0033)	0.02420	24	3	0.583 (0.00373)	0.02948
Wild *C. acutus*-Pinar del Río	2	2	1	0	0	2	1	0	0	2	1	0	0
*C. acutus*-Birama	27	19	1	0	0	7	2	0.286 (0.03856)	0.00055	26	1	0	0
*C. acutus*-Florida	12	–	–	–	–	11	1	0	0	12	1	0	0
*C. acutus*-Belize	12	–	–	–	–	12	1	0	0	12	1	0	0
*C. rhombifer*-Zapata	31	30	1	0	0	25	1	0	0	31	1	0	0

**Note:**

*N*, Total number of individuals per population; *n*, number of sequences used. The variance of the haplotype diversity is indicated between parentheses.

**Figure 2:**
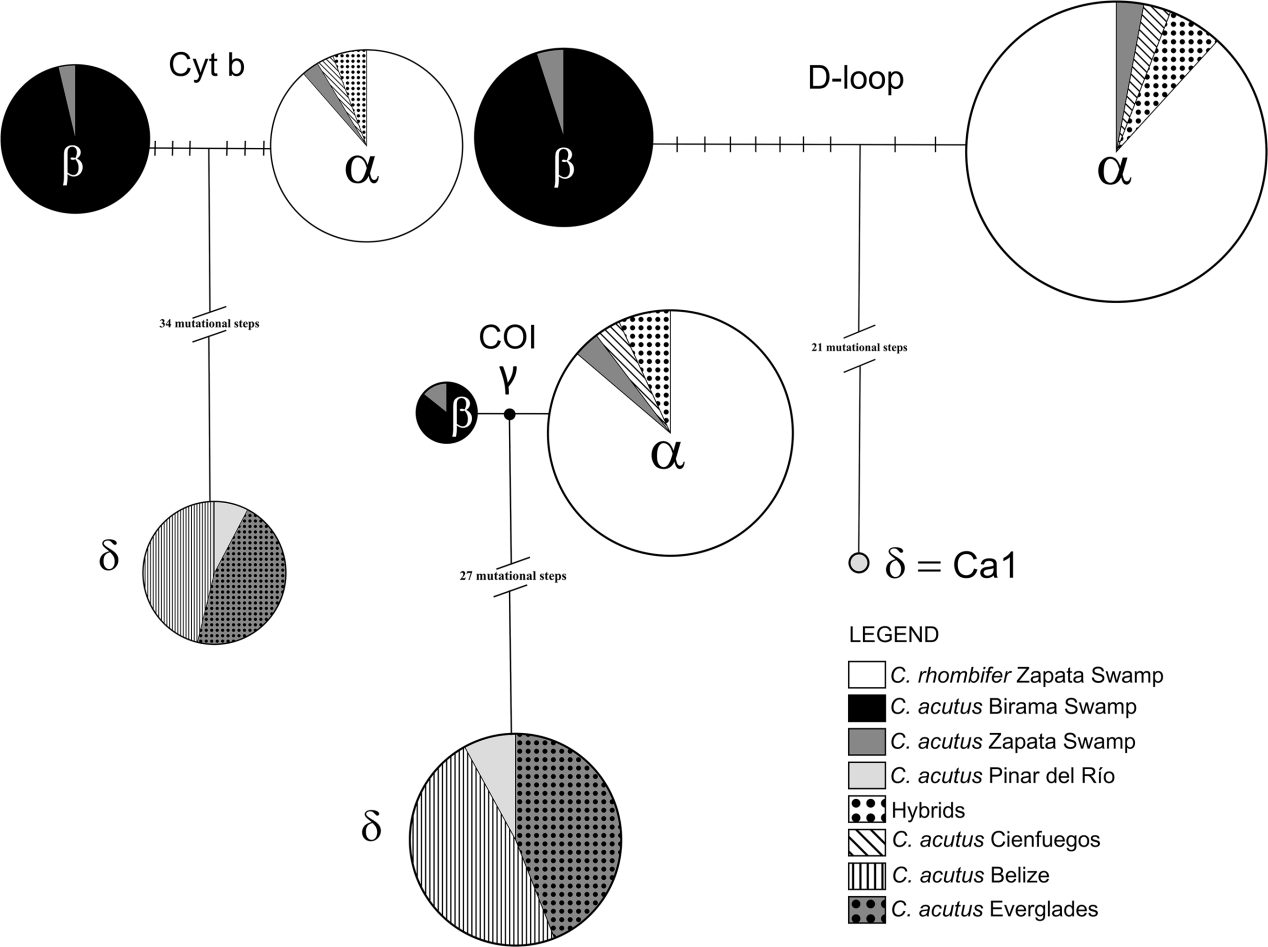
Haplotype networks generated for each mitochondrial DNA region for the *Crocodylus* populations included in the present study. Mutational steps between haplotypes are indicated by slashes. Greek letters correspond to the identifier of each sampled haplotype, following the order suggested in previous studies (Weaver et al., 2008).

Among the wild caught individuals, the most common haplotype was identical to the α haplotype (Weaver et al., 2008; Milián-García et al., 2015), which is shared by *C. rhombifer* (*n* = 30) and hybrids (*n* = 2) from Zapata Swamp, as well as for *C. acutus* from Cienfuegos (*n* = 1) and Zapata Swamp (*n* = 1) (Fig. 2). The second most common haplotype was identical to the β haplotype (Weaver et al., 2008; Milián-García et al., 2015), differing by nine steps from the α haplotype, and was found in *C. acutus* from Zapata Swamp (*n* = 1) and Birama Swamp (*n* = 19). The third most common haplotype was δ (identical to the Ca1 haplotype in Rodriguez et al. (2011); GenBank accession no. MH753361), which has been considered the most frequent haplotype in North and Central America (Fig. 2). It differs by 23 steps from the α haplotype and was found in all wild *C. acutus* from Pinar del Río (*n* = 2).

#### Cytochrome oxidase I

The COI fragment resolved into four haplotypes (Fig. 2) with an overall haplotype and nucleotide diversity per population ranging from 0.000–0.558 to 0.000–0.02420, respectively (Table 2). Similar to patterns at the D-loop, the most common haplotype was identical to the α haplotype and was shared among *C. rhombifer* (*n* = 25), *C. acutus* (*n* = 1), and hybrids (*n* = 2) from Zapata Swamp as well as for *C. acutus* from Cienfuegos (*n* = 1) (Fig. 2). The second most common haplotype δ (GenBank accession no. MH758763) was shared for *C. acutus* individuals from Pinar del Río (*n* = 2), Belize (*n* = 12), and Florida (*n* = 11). The third most common haplotype was identical to the β haplotype and was shared among *C. acutus* from Birama (*n* = 7), and Zapata Swamp (*n* = 1). The fourth mtDNA COI haplotype was unique to *C. acutus* from Birama (*n* = 1) and differed by just one step from the α and β haplotypes (GenBank accession no. MH758764).

#### Cytochrome b

Three mtDNA cytb haplotypes were recovered among the wild individuals sampled from Pinar del Río, Birama Swamp, Zapata Swamp, Belize, and Florida. Following the same pattern as for the D-loop, the most common haplotype was identical to the α haplotype and was shared among *C. rhombifer* (*n* = 31) and hybrids (*n* = 2) from Zapata Swamp, as well as for *C. acutus* from Cienfuegos (*n* = 1) and Zapata Swamp (*n* = 1) (Fig. 2). The second most common haplotype, δ, was shared for *C. acutus* from Pinar del Río (*n* = 2), Belize (*n* = 12), and Florida (*n* = 12) differing by 37 steps from the α haplotype (GenBank accession no. MH758762). The third haplotype was identical to the β haplotype and was shared among *C. acutus* from Birama (*n* = 26) and Zapata Swamps (*n* = 1), differing by six steps from the α haplotype. Overall haplotypic and nucleotide diversities ranged from 0.000–0.583 to 0.000–0.02948, respectively (Table 2).

The fully concatenated sequences revealed unique haplotypes for all Cuban samples for which all regions were successfully sequenced (Table S1). The AMOVA using the concatenated mtDNA sequences recovered significant levels of genetic structure, with a highest fraction distributed among (>99%), rather than within (<1%), the populations of Cuban and American crocodiles studied (*P* < 0.0001).

#### Captive population

Thirteen individuals with the D-loop α haplotype were identified in captivity, while two possessed the D-loop β haplotype and seven presented the D-loop δ haplotype. The other two individuals presented a fourth new haplotype differing by only one mutational step away from the δ haplotype (GenBank accession no. MH719199). When analyzing the COI fragment, captive individuals were distributed into three haplotypes. Twelve individuals presented the COI α haplotype, one the COI β haplotype and nine individuals shared the continental haplotype (δ). When analyzing the cytb, three haplotypes were recovered for the 24 captive individuals. A total of 13 individuals presented the cytb α haplotype, two exhibited the cytb β haplotype, while nine shared the same haplotype (δ) showed by the continental individuals.

#### Phylogenetic analysis

**Figure 3:**
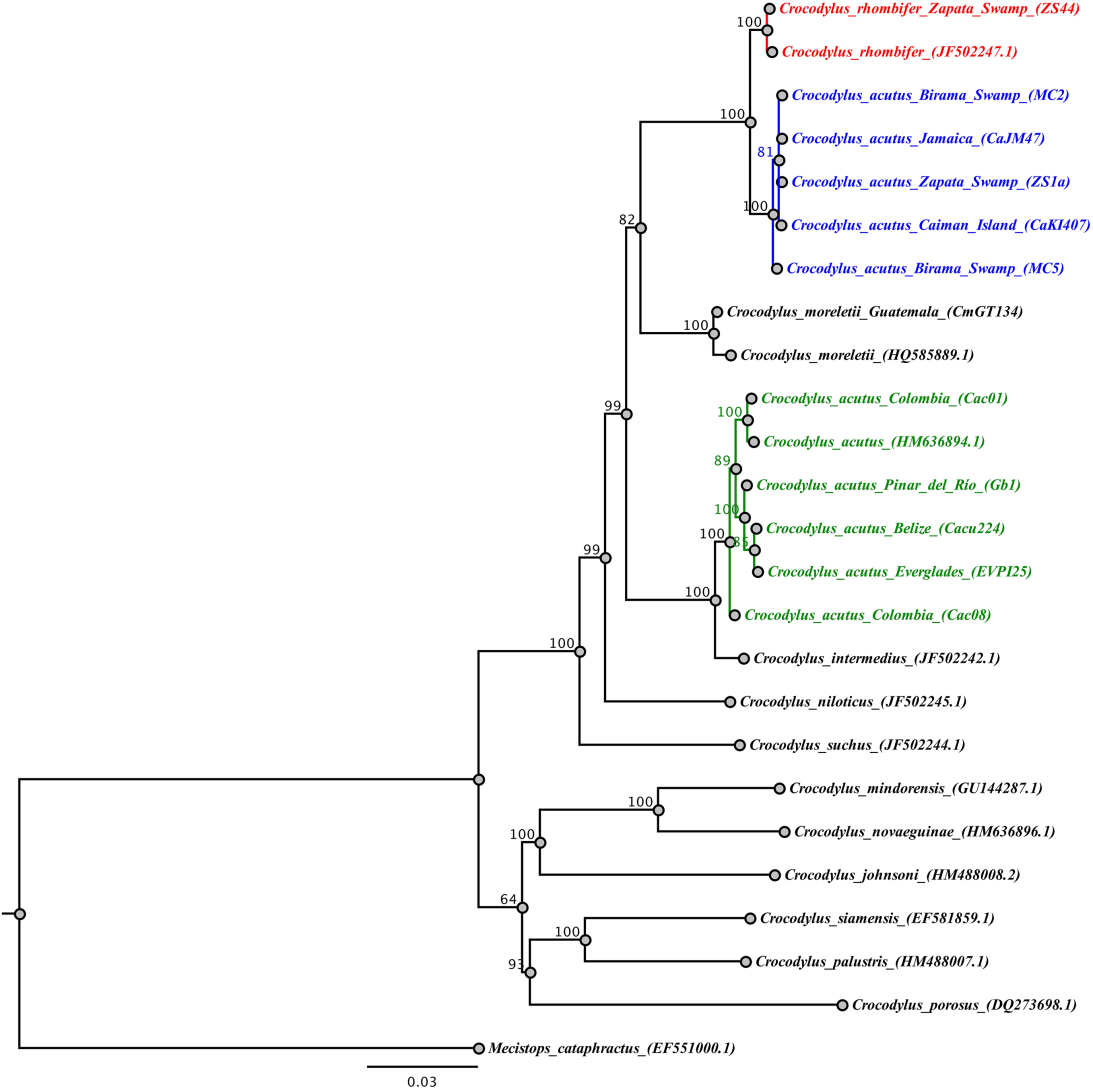
Phylogenetic tree based on Bayesian inference and the concatenated mtDNA dataset. The Cuban crocodile (*Crococylus rhombifer*) clade is highlighted in red, while the two clades for the American crocodile (*Crocodylus acutus*) are indicated in blue and green, respectively. The numbers on the nodes indicate posterior probability values expressed in percentage.

The analysis of the concatenated mtDNA regions for the wild-caught samples revealed that Cuban *Crocodylus* are split into three independent clades. Two of these correspond to the previous clades recovered for Antillean *C. acutus* (central and eastern Cuba, Jamaica, Cayman Islands) and *C. rhombifer* (Fig. 3) revealing a well-supported split (posterior probability = 1), and a sister relationship with *C. moreletti*. The third clade grouped wild *C. acutus* from western Cuba with *C. acutus* from continental South, Central, and North America, sister to *C. intermedius*. Tamura-Nei genetic distances revealed shallow divergence between Antillean *C. acutus* and *C. rhombifer* (0.4%), substantially lower than the value resolved between western and eastern *C. acutus* populations within Cuba (5.4%).

## Discussion

Recent studies have uncovered cryptic diversity across a diverse group of crocodylians (e.g., *C. niloticus*, *C. suchus*, *M. cataphractus*, *Osteolaemus*; McAliley et al., 2006; Eaton et al., 2009; Hekkala et al., 2011; Oaks, 2011; Shirley et al., 2014; Cunningham, Shirley & Hekkala, 2016). Consistent with previous findings (Milián-García et al., 2011, 2015, 2018), our results revealed a high degree of genetic differentiation within the American crocodile, *C. acutus*. Based on the most expansive geographic and mtDNA character sampling to date, we reconstructed *C. acutus* as paraphyletic, exhibiting two distinct lineages composed of Antillean and continental populations with high genetic divergence (5.4%). Moreover, these two lineages are more closely related to other species of crocodiles than they are to each other. Antillean *C. acutus* exhibits a close affiliation with *C. rhombifer* in a clade sister to *C. moreletii*. Conversely, continental *C. acutus* is most closely related to *C. intermedius*.

At a finer-scale, we found significant differentiation between haplotypes from western Cuba and the rest of the sampled populations, including Zapata Swamp and Birama Swamp. Interestingly, *C. acutus* haplotypes recovered from Pinar del Río province do not exhibit significant differentiation from continental *C. acutus*, and have individuals placed in the same clade (Fig. 3). This result further suggests that the western tip of Cuba—which is closer to the Yucatan peninsula than it is to Zapata Swamp—may either be a transition zone or an area of recent colonization from continental *C. acutus*. The fact that individuals sampled within the most western on-island *C. acutus* captive population share the continental haplotype is consistent with the hypothesis of a transition zone in the area. Most founders at the Sabanalamar captive population were captured locally back in the 1980s, while others were transported from Birama and Lanier Swamps (R. Rodríguez-Soberón, 2018, personal communication). The latter helps account for the admixture exhibited within the captive population. Our results also demonstrate that human-mediated translocation should no longer be a supported practice as it produces a mix of highly divergent genetic lineages.

The close genetic relationship between *C. rhombifer* and Antillean *C. acutus* based on mtDNA has been previously hypothesized to be a consequence of mitochondrial capture through hybridization, and *C. rhombifer* and *C. acutus* have been documented to hybridize in Zapata Swamp (Milián-García et al., 2015). Although karyological differences exist between *C. rhombifer* (2*n* = 30) and *C. acutus* (2*n* = 32) (Cohen & Gans, 1970), there is evidence of fertile hybrids (at least F1) between the species (E. Pérez-Fleitas, 2018, personal communication). However, most *C. acutus* populations in the Antilles have never been recorded to overlap in distribution with the Cuban crocodile (Tabet, 2010; Tabet et al., 2014; Brochu & Jiménez-Vázquez, 2014). For example, the American crocodile population in Birama Swamp, recognized as the second most extensive wetland in the insular Caribbean, is considered to be the largest and most protected population for the species in the Antilles (Tabet, 2010; Tabet et al., 2014). As such, mitochondrial capture through hybridization would be a feasible hypothesis to explain genetic similarity between *C. rhombifer* and Antillean *C. acutus* if the event occurred before the dispersion of *C. acutus* around the Cuban archipelago and the rest of the Greater Antilles. It would also imply that Antillean *C. acutus* with *C. rhombifer* captured-mitochondria would have higher fitness than the continental *C. acutus,* given the absence of the latter across the Antilles.

An alternative explanation for this pattern could be one of very recent divergence of the Antillean lineages from continental congeners coupled with the slow mtDNA mutation rate in crocodylians (Hekkala et al., 2011; Oaks, 2011). This hypothesis is consistent with the observation of low levels of genetic differentiation between *C. rhombifer* and Antillean *C. acutus* (Milián-García et al., 2011, 2015, 2018), albeit at a lower level than the average genetic distance between conspecifics within *Crocodylus* (∼1%; Hekkala et al., 2011). These findings, in tandem with observed morphological, ecological, and ethological characteristics (Cuvier, 1807; Targarona, 2013), suggest that *C. rhombifer* constitutes a unique lineage, but further study is warranted (see below).

These patterns reconstructed from mtDNA character data represent clear challenges for the current taxonomy of *C. acutus*. Combined with the results of previous studies (Weaver et al., 2008; Milián-García et al., 2011, 2015, 2018), we found evidence for a cryptic lineage of American crocodile currently found in Cuba and other localities in the Antilles. Moreover, the paraphyletic linages of *C. acutus* show a genetic distance (5.4%) similar to interspecific comparisons within *Crocodylus* and exhibit closer phylogenetic affinities with other species than each other, suggesting a formal taxonomic reassessment may be in order. Yet it is imperative to highlight that this work was limited to insights from mitochondrial DNA character data; additional analyses using nuclear data are essential for resolving taxonomic uncertainty in this system and to more directly test phylogeographic hypotheses. That said, our results do have immediate relevance for conservation, as the cryptic diversity uncovered here and elsewhere is not currently accounted for in status assessments and management plans for new world *Crocodylus*.

## Conclusions

Overall, the reconstructed paraphyly, distinct phylogenetic affinities and high genetic divergence between Antillean and continental *C. acutus* populations reinforce the need for a formal taxonomic reassessment. Such an endeavor would benefit from a nuclear genome-wide study of *Crocodylus*, as has been demonstrated in other systems that exhibit a complex history and high prevalence of cryptic diversity (e.g., Galapagos tortoise, *Chelonoidis* sp.; Miller et al., 2018). This work benefited from the most comprehensive geographic and character data sampling conducted to date for *C. acutus* and other congenerics. Importantly, the inclusion, for the first time, of samples from the western population of the American crocodile in Cuba revealed evidence for continental mtDNA haplotypes in the Antilles, suggesting this area may constitute a transition zone between distinct lineages of *C. acutus*. More broadly, detection of cryptic diversity has important implications for conservation. Although *C. acutus* population sizes have been increasing since CITES protections have been in place (Platt, Rainwater & Nichols, 2004), assessments to date have not evaluated the status of Antillean populations as potentially constituting an independently evolving lineage of New World crocodiles. Likewise, cryptic diversity may be reflective of ongoing evolutionary processes as closely related species and populations adapt to changing environments (Bickford et al., 2007), punctuating the need for formal characterization to resolve taxonomic uncertainty and inform conservation planning (Struck et al., 2018), in this case for the American and Cuban crocodiles.

## Supplemental Information

10.7717/peerj.5836/supp-1 Supplemental Information 1 Mitochondrial regions sequenced per sample in the present study, including the morphologic identification and sample locality.

10.7717/peerj.5836/supp-2 Supplemental Information 2 Reference mitogenomes for Crocodylus.

10.7717/peerj.5836/supp-3 Supplemental Information 3 Raw data: COI Belize Crocodylus.

10.7717/peerj.5836/supp-4 Supplemental Information 4 Raw data: Cyt b Belize Crocodylus.

10.7717/peerj.5836/supp-5 Supplemental Information 5 Raw data: COI Frorida Crocodylus.

10.7717/peerj.5836/supp-6 Supplemental Information 6 Raw data: Cyt b Frorida Crocodylus.

10.7717/peerj.5836/supp-7 Supplemental Information 7 Raw data: COI Birama Crocodylus.

10.7717/peerj.5836/supp-8 Supplemental Information 8 Raw data: Dloop Birama Crocodylus.

10.7717/peerj.5836/supp-9 Supplemental Information 9 Raw data: Cyt b Birama Crocodylus.

10.7717/peerj.5836/supp-10 Supplemental Information 10 Raw data: COI Pinar Del Rio Crocodylus.

10.7717/peerj.5836/supp-11 Supplemental Information 11 Raw data: Dloop Pinar Del Rio Crocodylus.

10.7717/peerj.5836/supp-12 Supplemental Information 12 Raw data: Cyt b Pinar Del Rio Crocodylus.

10.7717/peerj.5836/supp-13 Supplemental Information 13 Raw data: COI Zapata Crocodylus.

10.7717/peerj.5836/supp-14 Supplemental Information 14 Raw data: Dloop Zapata Crocodylus.

10.7717/peerj.5836/supp-15 Supplemental Information 15 Raw data: Cyt b Zapata Crocodylus.

10.7717/peerj.5836/supp-16 Supplemental Information 16 Raw data: COI Crocodylus Captive.

10.7717/peerj.5836/supp-17 Supplemental Information 17 Raw data: Dloop Crocodylus Captive.

10.7717/peerj.5836/supp-18 Supplemental Information 18 Raw data: Cyt b Crocodylus Captive.

## Additional Information and Declarations

### Field Study Permissions

The following information was supplied relating to field study approvals (i.e., approving body and any reference numbers): All samples were collected and transported in accordance with an agreement established between the Faculty of Biology at the University of Havana and the National Enterprise for the Protection of Flora and Fauna in Cuba, and CITES export and import permits, C002135 and E-00134/14, respectively.

### Data Availability

The following information was supplied regarding data availability: GenBank accession no. MH719199, MH753361, MH758762–MH758764.

## References

[ref-1] Bickford D, Lohman DJ, Sodhi NS, Ng PKL, Meier R, Winker K, Ingram KK, Das I (2007). Cryptic species as a window on diversity and conservation. Trends in Ecology & Evolution.

[ref-39] Bloor P, Ibáñez C, Viloria-Lagares TA (2015). Mitochondrial DNA analysis reveals hidden genetic diversity in captive populations of the threatened American crocodile (*Crocodylus acutus*) in Colombia. Ecology and Evolution.

[ref-2] Brochu CA, Jiménez-Vázquez O (2014). Enigmatic crocodyliforms from the early miocene of Cuba. Journal of Vertebrate Paleontology.

[ref-3] Brochu CA, McEachran JD (2000). Phylogenetic relationships and divergence timing of crocodylus based on morphology and the fossil record. Copeia.

[ref-45] Clement M, Posada D, Crandall KA (2000). TCS: a computer program to estimate gene genealogies. Molecular Ecology.

[ref-4] Cohen MM, Gans C (1970). The chromosomes of the order crocodilia. Cytogenetic and Genome Research.

[ref-5] Cunningham SW, Shirley MH, Hekkala ER (2016). Fine scale patterns of genetic partitioning in the rediscovered African crocodile, *Crocodylus suchus* (Saint-Hilaire 1807). PeerJ.

[ref-6] Cuvier G (1807). Sur les differentes especes de crocodiles vivantes et sur leurs caracteres distinctifs. Annales du Muséum d’histoire Naturelle.

[ref-7] Eaton MJ, Martin A, Thorbjarnarson J, Amato G (2009). Species-level diversification of African dwarf crocodiles (Genus Osteolaemus): a geographic and phylogenetic perspective. Molecular Phylogenetics and Evolution.

[ref-8] Excoffier L, Lischer HEL (2010). Arlequin suite ver 3.5: a new series of programs to perform population genetics analyses under Linux and Windows. Molecular Ecology Resources.

[ref-9] Excoffier L, Smouse PE, Quattro JM (1992). Analysis of molecular variance inferred from metric distances among DNA haplotypes: application to human mitochondrial DNA restriction data. Genetics.

[ref-41] Feng G, Wu X, Yan P, Li X (2010). Two complete mitochondrial genomes of *Crocodylus* and implications for crocodilians phylogeny. Amphibia-Reptilia.

[ref-10] Grigg G, Kirshner D (2015). Biology and evolution of Crocodylians.

[ref-11] Hekkala ER, Platt SG, Thorbjarnarson JB, Rainwater TR, Tessler M, Cunningham SW, Twomey C, Amato G (2015). Integrating molecular, phenotypic and environmental data to elucidate patterns of crocodile hybridization in Belize. Royal Society Open Science.

[ref-12] Hekkala E, Shirley MH, Amato G, Austin JD, Charter S, Thorbjarnarson J, Vliet KA, Houck ML, Desalle R, Blum MJ (2011). An ancient icon reveals new mysteries: mummy DNA resurrects a cryptic species within the Nile crocodile. Molecular Ecology.

[ref-13] Kearse M, Moir R, Wilson A, Stones-Havas S, Cheung M, Sturrock S, Buxton S, Cooper A, Markowitz S, Duran C, Thierer T, Ashton B, Meintjes P, Drummond A (2012). Geneious basic: an integrated and extendable desktop software platform for the organization and analysis of sequence data. Bioinformatics.

[ref-14] Kessing B, Croom H, Martin A, McIntosh C, Mcmillan WO, Palumbi S (1989). The Simple Fool’s Guide to PCR.

[ref-15] Kumar S, Stecher G, Tamura K (2016). MEGA7: molecular evolutionary genetics analysis version 7.0 for bigger datasets. Molecular Biology and Evolution.

[ref-40] Li Y, Wu X, Ji X, Yan P, Amato G (2007). The complete mitochondrial genome of salt-water crocodile (*Crocodylus porosus*) and phylogeny of crocodilians. Journal of Genetics and Genomics.

[ref-44] Librado P, Rozas J (2009). DnaSP v5: a software for comprehensive analysis of DNA polymorphism data. Bioinformatics.

[ref-16] Man Z, Yishu W, Peng Y, Xiaobing W (2011). Crocodilian phylogeny inferred from twelve mitochondrial protein-coding genes, with new complete mitochondrial genomic sequences for *Crocodylus acutus* and *Crocodylus novaeguineae*. Molecular Phylogenetics and Evolution.

[ref-17] McAliley LR, Willis RE, Ray DA, White PS, Brochu CA, Densmore LD (2006). Are crocodiles really monophyletic?—Evidence for subdivisions from sequence and morphological data. Molecular Phylogenetics and Evolution.

[ref-18] Meganathan PR, Dubey B, Batzer MA, Ray DA, Haque I (2010). Molecular phylogenetic analyses of genus *Crocodylus* (Eusuchia, Crocodylia, Crocodylidae) and the taxonomic position of *Crocodylus porosus*. Molecular Phylogenetics and Evolution.

[ref-19] Meredith RW, Hekkala ER, Amato G, Gatesy J (2011). A phylogenetic hypothesis for *Crocodylus* (Crocodylia) based on mitochondrial DNA: evidence for a trans-Atlantic voyage from Africa to the New World. Molecular Phylogenetics and Evolution.

[ref-20] Milián-García Y, Castellanos-Labarcena J, Russello MA, Amato G (2018). Mitogenomic investigation reveals a cryptic lineage of *Crocodylus* in Cuba. Bulletin of Marine Science.

[ref-21] Milián-García Y, Ramos-Targarona R, Pérez-Fleitas E, Sosa-Rodríguez G, Guerra-Manchena L, Alonso-Tabet M, Espinosa-López G, Russello MA (2015). Genetic evidence of hybridization between the critically endangered Cuban crocodile and the American crocodile: implications for population history and in situ/ex situ conservation. Heredity.

[ref-22] Milián-García Y, Venegas-Anaya M, Frias-Soler R, Crawford AJ, Ramos-Targarona R, Rodríguez-Soberón R, Alonso-Tabet M, Thorbjarnarson J, Sanjur OI, Espinosa-López G, Bermingham E (2011). Evolutionary history of Cuban crocodiles *Crocodylus rhombifer* and *Crocodylus acutus* inferred from multilocus markers. Journal of Experimental Zoology. Part A: Ecological Genetics and Physiology.

[ref-23] Miller JM, Quinzin MC, Edwards DL, Eaton DAR, Jensen EL, Russello MA, Gibbs JP, Tapia W, Rueda D, Caccone A (2018). Genome-wide assessment of diversity and divergence among extant galapagos giant tortoise species. Journal of Heredity.

[ref-24] Múrias dos Santos A, Cabezas MP, Tavares AI, Xavier R, Branco M (2016). tcsBU: a tool to extend TCS network layout and visualization. Bioinformatics.

[ref-43] Nei M (1987). Molecular Evolutionary Genetics.

[ref-25] Oaks JR (2011). A time-calibrated species tree of crocodylia reveals a recent radiation of the true crocodiles. Evolution.

[ref-26] Palumbi S, Hillis DM, Moritz C, Mable BK (1996). Nucleic acids II: the polymerase chain reaction. Molecular Systematics.

[ref-27] Platt SG, Rainwater TR, Nichols S (2004). A recent population assessment of the American crocodile (*Crocodylus acutus*) in Turneffe Atoll, Belize. Herpetological Bulletin.

[ref-28] Posada D, Crandall KA (1998). MODELTEST: testing the model of DNA substitution. Bioinformatics.

[ref-29] Ray DA, Dever JA, Platt SG, Rainwater TR, Finger AG, McMurry ST, Batzer MA, Barr B, Stafford PJ, McKnight J, Densmore LD (2004). Low levels of nucleotide diversity in *Crocodylus moreletii* and evidence of hybridization with *C. acutus*. Conservation Genetics.

[ref-38] Rodriguez D, Forstner MRJ, Moler PE, Wasilewski JA, Cherkiss MS, Iii LDD (2011). Effect of human-mediated migration and hybridization on the recovery of the American crocodile in Florida (USA). Conservation Genetics.

[ref-30] Ronquist F, Huelsenbeck JP (2003). MrBayes 3: Bayesian phylogenetic inference under mixed models. Bioinformatics.

[ref-31] Rossi NA (2016). Population genetic structure and reproductive ecology of *Crocodylus* across local and regional scales.

[ref-32] Shirley MH, Vliet KA, Carr AN, Austin JD (2014). Rigorous approaches to species delimitation have significant implications for African crocodilian systematics and conservation. Proceedings of the Royal Society of London B: Biological Sciences.

[ref-42] Srikulnath K, Thongpan A, Suputtitada S, Apisitwanich S (2012). New haplotype of the complete mitochondrial genome of *Crocodylus siamensis* and its species-specific DNA markers: distinguishing *C. siamensis* from *C. porosus* in Thailand. Molecular Biology Reports.

[ref-33] Struck TH, Feder JL, Bendiksby M, Birkeland S, Cerca J, Gusarov VI, Kistenich S, Larsson K-H, Liow LH, Nowak MD, Stedje B, Bachmann L, Dimitrov D (2018). Finding evolutionary processes hidden in cryptic species. Trends in Ecology & Evolution.

[ref-34] Tabet MA (2010). Comportamiento del cocodrilo americano (*Crocodylus acutus*) en el refugio de fauna monte Cabaniguan Cuba. http://purl.org/dc/dcmitype/Text.

[ref-35] Tabet MA, Targarona RR, Soberón RR, Thorbjarnarson J, Ferrer JB, Álvarez VB (2014). Los Crocodylia de Cuba.

[ref-36] Targarona RR (2013). Ecologia y conservación del cocodrilo cubano (Crocodylus rhombifer) en la “Ciénaga de Zapata,” Cuba. http://purl.org/dc/dcmitype/Text.

[ref-37] Weaver JP, Rodriguez D, Venegas-Anaya M, Cedeño-Vázquez JR, Forstner MRJ, Densmore LD (2008). Genetic characterization of captive Cuban crocodiles (*Crocodylus rhombifer*) and evidence of hybridization with the American crocodile (*Crocodylus acutus*). Journal of Experimental Zoology Part A: Ecological Genetics and Physiology.

